# The Sir2-Sum1 Complex Represses Transcription Using Both Promoter-Specific and Long-Range Mechanisms to Regulate Cell Identity and Sexual Cycle in the Yeast *Kluyveromyces lactis*

**DOI:** 10.1371/journal.pgen.1000710

**Published:** 2009-11-06

**Authors:** Meleah A. Hickman, Laura N. Rusche

**Affiliations:** 1 Institute for Genome Sciences and Policy, Duke University, Durham, North Carolina, United States of America; 2 University Program in Genetics and Genomics, Duke University, Durham, North Carolina, United States of America; 3 Department of Biochemistry, Duke University Medical Center, Durham, North Carolina, United States of America; University of California San Francisco, United States of America

## Abstract

Deacetylases of the Sir2 family regulate lifespan and response to stress. We have examined the evolutionary history of Sir2 and Hst1, which arose by gene duplication in budding yeast and which participate in distinct mechanisms of gene repression. In *Saccharomyces cerevisiae*, Sir2 interacts with the SIR complex to generate long-range silenced chromatin at the cryptic mating-type loci, *HMLα* and *HMR* ***a***. Hst1 interacts with the SUM1 complex to repress sporulation genes through a promoter-specific mechanism. We examined the functions of the non-duplicated Sir2 and its partners, Sir4 and Sum1, in the yeast *Kluyveromyces lactis*, a species that diverged from *Saccharomyces* prior to the duplication of Sir2 and Hst1. KlSir2 interacts with both KlSir4 and KlSum1 and represses the same sets of target genes as ScSir2 and ScHst1, indicating that Sir2 and Hst1 subfunctionalized after duplication. However, the KlSir4-KlSir2 and KlSum1-KlSir2 complexes do not function as the analogous complexes do in *S. cerevisiae*. KlSir4 contributes to an extended repressive chromatin only at *HMLα* and not at *HMR* ***a***. In contrast, the role of KlSum1 is broader. It employs both long-range and promoter-specific mechanisms to repress cryptic mating-type loci, cell-type–specific genes, and sporulation genes and represents an important regulator of cell identity and the sexual cycle. This study reveals that a single repressive complex can act through two distinct mechanisms to regulate gene expression and illustrates how mechanisms by which regulatory proteins act can change over evolutionary time.

## Author Summary

Sir2 deacetylases are found in organisms ranging from bacteria to mammals. Sir2 from the yeast *Saccharomyces cerevisiae* deacetylates histones and is part of the SIR complex that spreads across chromatin to repress gene expression. A related histone deacetylase, Hst1, interacts with a DNA–binding protein, Sum1, to repress genes in a promoter-specific manner. Hst1 and Sir2 are paralogs, arising from a duplication about 100 million years ago. To understand how Sir2 and Hst1 have diverged, as well as to investigate the evolutionary relationship between spreading and non-spreading mechanisms of gene repression, we have characterized the function of a non-duplicated Sir2 from the yeast *Kluyveromyces lactis*, a species that diverged from *Saccharomyces* prior to this duplication. We found that KlSir2 is part of both the SIR and SUM1 complexes, indicating that the ancestral Sir2 had both Sir2- and Hst1-like properties. Interestingly, we found that, in *K. lactis*, the Sir2-Sum1 complex not only uses a promoter-specific mechanism to repress the same sets of genes as *S. cerevisiae*, it also forms extended chromatin structures to repress gene transcription. Our results illustrate how mechanisms by which regulatory proteins act can change over evolutionary time.

## Introduction

Deacetylases of the Sir2 family are key regulators of lifespan and stress resistance in many organisms ranging from yeast to humans [1]. These enzymes couple deacetylation with hydrolysis of NAD^+^ and consequently their activity is linked to the metabolic state of the cell [2]. Despite having a well-conserved enzymatic activity, Sir2 family members act on a wide variety of substrates and serve a diverse set of biological functions [3],[4]. To explore the process by which Sir2 deacetylases have diversified, we examined the evolutionary history of two family members from budding yeast, Sir2 and Hst1 [5],[6], which arose in a whole-genome duplication [7],[8],[9], yet have distinct functions.

Gene duplication is an important force in evolution because it allows variation to occur without compromising the original function of the gene. Preservation of duplicate genes, or paralogs, is proposed to occur through at least two mechanisms, neofunctionalization and subfunctionalization. In the neofunctionalization model, one duplicate retains the original function, leaving the other gene free of selective constraint and able to evolve a new function [10]. Alternatively, in the subfunctionalization model, if the ancestral gene had multiple functions, duplicated genes could each lose one of the original functions and together retain the entire set of ancestral functions [11]. Only a few studies have characterized the path by which paralogs have diverged [12],[13],[14],[15]. To investigated how Sir2 and Hst1 diverged, we have characterized the function of a representative non-duplicated Sir2 from *Kluyveromyces lactis*, a budding yeast species that diverged from *S. cerevisiae* prior to the whole-genome duplication [16].

The functions of Sir2 and Hst1 in *S. cerevisiae* are well understood. Sir2 interacts with the histone-binding proteins Sir3 and Sir4, and together these proteins generate an extended silenced domain at the telomeres and cryptic mating-type loci, *HMLα* and *HMR* ***a*** [17]. The *HM* loci are flanked by silencers that recruit Sir proteins through DNA binding proteins to initiate the formation of silenced chromatin. The telomere repeats also recruit Sir proteins. Sir2, Sir3, and Sir4 spread from sites of recruitment through a sequential deacetylation mechanism that is independent of DNA sequence [18],[19],[20]. Sir2 deacetylates nearby nucleosomes, creating high affinity binding sites for Sir3 and Sir4, which bind preferentially to deacetylated tails of histones H3 and H4. Sir3 and Sir4 then recruit additional Sir2 to newly deacetylated nucleosomes. As Sir proteins spread, they generate a specialized chromatin structure that is restrictive to transcription.

Unlike Sir2, Hst1 does not spread. It is part of the SUM1 complex that represses over fifty genes that are involved in sporulation, NAD^+^ biosynthesis, and *α*-cell identity [21],[22],[23],[24]. Sum1 is a DNA binding protein that associates with a conserved sequence, the middle sporulation element, found in the promoters of target genes [21],[23],[25]. Hst1 deacetylates the tails of histones H3 and H4 [26],[27], and this deacetylation is thought to be important for its repressive function. The third member of the complex, Rfm1, mediates the interaction between Sum1 and Hst1 [22].

Genes regulated by Sir2 and Hst1 are critical to cell identity as well as the sexual cycle, and consequently these deacetylases have the potential to coordinate the timing of the life cycle with NAD^+^ availability. Hst1 plays a role in cell-type identity by repressing several *α*-specific genes [24]. Hst1 also represses a number of mid-sporulation genes, and this repression must be relieved for completion of the sexual cycle [23]. The mating-type of haploid yeast cells, which can be **a** or *α*, is determined by the *MAT* locus, which encodes transcription factors that regulate cell-type specific genes [28]. These transcription factors are also encoded at *HMLα* and *HMR* ***a***, but are silenced by the SIR complex and serve as repositories for mating-type switching. Sir2 maintains cell identity by preventing the cell from simultaneously expressing both **a**- and *α*-specific transcription factors.

Compared to ScSir2 and ScHst1, the biological function of the non-duplicated KlSir2 is less understood. KlSir2 is thought to have properties similar to both Sir2 and Hst1, as it complements both *sir2Δ* and *hst1Δ* mutations in *S. cerevisiae* [26],[29]. In *K. lactis*, KlSir2 represses the *HM* loci [30],[31], and a *sir2Δ* mutation results in reduced mating and sporulation defects [29]. Prior to this study, it was not known whether KlSir2 regulates sporulation genes as ScHst1 does.

Few studies have investigated silencing in *K. lactis*, yet the mechanism differs substantially from that in *S. cerevisiae*. KlSir2 and the histone binding protein KlSir4 contribute to the silencing of *HMLα* [30],[31]. However, there is no distinct Sir3 protein in *K. lactis*. Additionally, the silencer elements that recruit silencing factors are not conserved between *K. lactis* and *S. cerevisiae* [32]. Silencers in *S. cerevisiae* consist of binding sites for ORC, Rap1, and Abf1, whereas in *K. lactis*, binding sites for these factors have not been identified at the *HM* loci. Instead the only defined silencer consists of a KlReb1 binding site and two other uncharacterized DNA sequences [32].

In this study, we examined the functions of the non-duplicated KlSir2 and found that it interacts with both KlSir4 and KlSum1. However, the SIR and SUM1 complexes in *K. lactis* do not function exactly as the analogous complexes do in *S. cerevisiae*. The KlSum1-KlSir2 complex contributes to silencing at both *HM* loci as well as sporulation and cell-type specific genes and achieves repression by both long-range and promoter-specific mechanisms. In contrast, KlSir4 only contributes to silenced chromatin at *HMLα*, but not at *HMR* ***a***. This study enhances our understanding of the process by which duplicated genes diverge and provides insights into the connections between promoter-specific and regional silencing.

## Results

### KlSir2 physically associates with both KlSir4 and KlSum1

**Figure 1:**
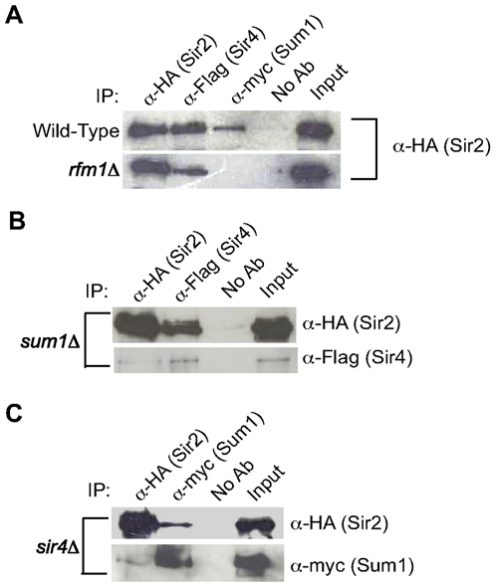
KlSir2 co-precipitates with KlSir4 and KlSum1. (A) KlSir2-HA, KlSir4-Flag or myc-KlSum1 was precipitated from a lysate prepared from wild-type (LRY2285) or *rfm1Δ* (LRY2528) strains, and the precipitated material was examined by immunoblotting with an antibody against the HA tag to detect KlSir2-HA. The input represents 33% of the IP. (B) KlSir2-HA or KlSir4-Flag was immunoprecipitated from a *sum1*Δ strain (LRY2158), and the precipitated material was examined with an antibody against the HA tag to detect KlSir2-HA or the Flag tag to detect KlSir4-Flag. (C) KlSir2-HA or myc-KlSum1 was immunoprecipitated from a *sir4*Δ strain (LRY2282), and the precipitated material was examined with an antibody against the HA tag to detect KlSir2-HA or the myc tag to detect myc-KlSum1.

To determine whether the non-duplicated KlSir2 has functions analogous to both ScSir2 and ScHst1, we first identified its binding partners in *K. lactis* (described in Table 1). If KlSir2 functions similarly to ScSir2, it should associate with KlSir4, and if it has a function analogous to ScHst1 it should associate with KlSum1. Trans-species complementation experiments previously demonstrated that KlSir2 associates with both ScSir4 and ScSum1 in *S. cerevisiae* [26], suggesting that analogous interactions occur in *K. lactis*. We created a *K. lactis* strain with alleles of *KlSIR2*-HA, *KlSIR4*-Flag and myc-*KlSUM1* integrated at their chromosomal locations. All three tagged proteins were detectable by immunoblotting (Figure 1) and maintained wild-type function, as assessed by RT-PCR analysis of genes repressed by these proteins (data not shown).

**Table 1:** Overview of *K. lactis* genes described in this study.

Common Name	*K. lactis* systematic name	*S. cerevisiae* homolog	Conservation^1^	Biological Function in *S. cerevisiae*
*KlSIR2*	*KLLA0F14663g*	*ScSIR2* 2	**56** (78)	Silences *HML*, *HMR*, telomeres, and the rDNA locus, in complex with Sir4 and Sir3
		*ScHST1* 2	**63** (84)	Repressor of middle sporulation-specific genes, in complex with Rfm1 and Sum1
*KlSUM1*	*KLLA0C14696g*	*ScSUM1*	**33** (59)	Repressor of middle sporulation-specific genes, in complex with Rfm1 and Hst1
*KlRFM1*	*KLLA0C07062g*	*ScRFM1*	**36** (63)	Repressor of middle sporulation-specific genes, in complex with Hst1 and Sum1
*KlSIR4* 3	*KLLA0F14320g*	See Figure S1		Silences *HML*, *HMR* and telomeres, in complex with Sir2 and Sir3
*KlASF2* 3	*KLLA0F13998g*	See Figure S1		Anti-silencing protein that causes derepression of silent loci when overexpressed

1 Percent identity (percent similar), calculated from FASTA sequence alignments.

2 *SIR2* and *HST1* are a duplicate gene pair, duplicated in the whole-genome duplication.

3 *SIR4* and *ASF2* are a tandem duplicate gene pair, duplicated prior to the whole-genome duplication.

If KlSir2 associates with both KlSir4 and KlSum1, it should co-precipitate with these proteins, and indeed, KlSir2 did co-precipitate with both KlSir4 and KlSum1 (Figure 1A). In *S. cerevisiae*, the association of ScSum1 with ScHst1 requires ScRfm1 [22]. To determine if Rfm1 mediates the interaction between Sum1 and Sir2 in *K. lactis*, we examined whether the co-precipitation between KlSir2 and KlSum1 persisted in the absence of KlRfm1. There was no observable co-precipitation between KlSir2 and KlSum1 in an *rfm1*Δ strain (Figure 1A), suggesting that the architecture of the SUM1 complex is conserved between *S. cerevisiae* and *K. lactis*.

Given the association of KlSir2 with both KlSir4 and KlSum1, all three proteins might be part of a stable complex. However, a co-precipitation between KlSir4 and KlSum1 was not detected (data not shown), although we could not distinguish whether this result reflected the absence of a complex containing KlSir4 and KlSum1 or simply its instability. Nevertheless, if this complex does exist, the components are not mutually dependent on one another for association, as KlSir2 and KlSir4 still co-precipitated in the absence of KlSum1 (Figure 1B) and KlSir2 and KlSum1 co-precipitated in the absence of KlSir4 (Figure 1C). Therefore, KlSir2 forms independent associations with both KlSir4 and KlSum1, a finding consistent with KlSir2 having functions analogous to those of both ScSir2 and ScHst1.

### KlSir2, KlSir4, and KlSum1 repress *HMLα*

**Figure 2:**
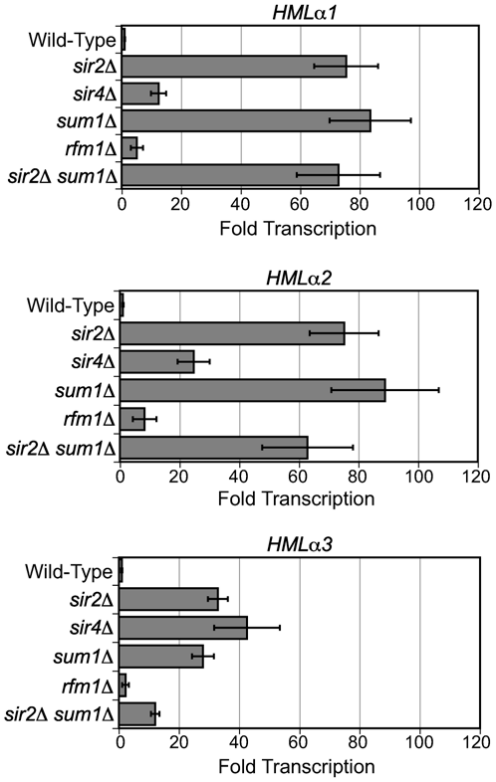
KlSir2, KlSir4, and KlSum1 silence the cryptic mating-type locus *HMLα*. Quantitative RT–PCR analysis of *HMLα1*, *HMLα2* and *HMLα3* mRNA in wild-type (CK213), *sir2*Δ (SAY569), *sir4*Δ (LRY2038), *sum1*Δ (LRY2035), *rfm1*Δ (LRY2528), and *sir2*Δ *sum1*Δ (LRY2533) strains. The amount of cDNA was first normalized to the control locus *ACT1*. The values shown here represent the relative amount of cDNA for each deletion strain compared to the wild-type strain. Error bars represent the SEM.

We next investigated whether the Sir4-Sir2 and Sum1-Sir2 complexes have the same repressive functions in *K. lactis* as they do in *S. cerevisiae*. If these functions are conserved, deletion of *KlSIR4* should derepress the *HM* loci, deletion of *KlSUM1* should derepress mid-sporulation genes, and deletion of *KlSIR2* should derepress both *HM* loci and mid-sporulation genes. We first examined silencing at *HMLα*, which is known to be repressed by KlSir2 and KlSir4 [30],[31]. To extend this previous result and address the role of KlSum1 at *HMLα*, we isolated RNA from *MAT* ***a*** wild-type, *sir2*Δ, *sir4*Δ, *sum1*Δ, and *rfm1*Δ strains and examined the expression of *HMLα1*, *HMLα2* and *HMLα3* by quantitative RT-PCR. All three genes were significantly derepressed in the absence of KlSir2 and modestly derepressed in the absence of KlSir4 (Figure 2), consistent with previous reports. Surprisingly, deletion of KlSum1 resulted in derepression of *HMLα* to a similar extent as observed in the *sir2*Δ strain. In contrast to KlSum1, deletion of KlRfm1 had very little effect on the transcription of *HMLα*. This result suggests that KlSir2 does not require KlRfm1 to act at *HMLα* and therefore may act independently of KlSum1. In this case, a *sir2*Δ *sum1*Δ double deletion might disrupt silencing to a greater extent than either single deletion. However, there was no difference in transcription of *HMLα* in a *sir2*Δ *sum1*Δ strain compared to a *sir2*Δ or *sum1*Δ strain (Figure 2).

To confirm that these phenotypes resulted from the deletions of the intended genes, plasmids expressing the wild-type *KlSIR2*, *KlSIR4* and *KlSUM1* genes were introduced into the corresponding deletion strains. In all cases, repression was restored (data not shown). These results reveal that KlSum1, in addition to KlSir2 and KlSir4, contributes to the silencing of *HMLα*. Thus, KlSum1 behaves differently than its ortholog in *S. cerevisiae*, as the deletion of ScSum1 does not alter the expression of *ScHMLα* [33].

It is interesting to note that in both the *sir2*Δ and *sum1*Δ strains the induction of *HMLα3* was modest compared to *HMLα1* or *HMLα2*, suggesting that *HMLα3* may be regulated differently than the other two genes at *HMLα*. The *α3* gene, which is specific to *Kluyveromyces*, is proposed to be a MULE family DNA transposase [34] and is required for mating [30].

The modest derepression of the *HMLα* locus observed in the *sir4*Δ strain suggested that another protein might compensate for KlSir4 in its absence. The *SIR4* gene was duplicated in tandem prior to the whole-genome duplication, and each of the tandem duplicates was retained as a single gene after the whole-genome duplication [7]. This ancient duplicate of Sir4, Asf2 (Anti-Silencing Factor 2), reduces silencing when over-expressed in *S. cerevisiae* [35]. The *SIR4* and *ASF2* genes are rapidly evolving, making it difficult to determine which *K. lactis* gene is orthologous to which *S. cerevisiae* gene (Figure S1). Gene *KLLAOF14320g* has been designated *KlSIR4* based on functional studies [31], and therefore we refer to the other gene (*KLLA0F13398g*) as *KlASF2*. To determine whether its common ancestry with KlSir4 enables KlAsf2 to silence *HMLα* in the absence of KlSir4, we constructed both *asf2*Δ and *asf2*Δ *sir4*Δ strains and examined expression of the *HMLα* genes. The lack of KlAsf2 resulted in the further repression of all three genes to less than one-tenth the level of the wild-type strain, and the double deletion of *asf2*Δ and *sir4*Δ resembled the single *sir4*Δ deletion (Figure S2). Therefore, *KlASF2* does not have a *SIR4*-like function. In fact, *KlASF2*, like *ScASF2*, is antagonistic to silencing.

### KlSir2, KlSir4, and KlSum1 spread across *HMLα* but not *MATα*

**Figure 3:**
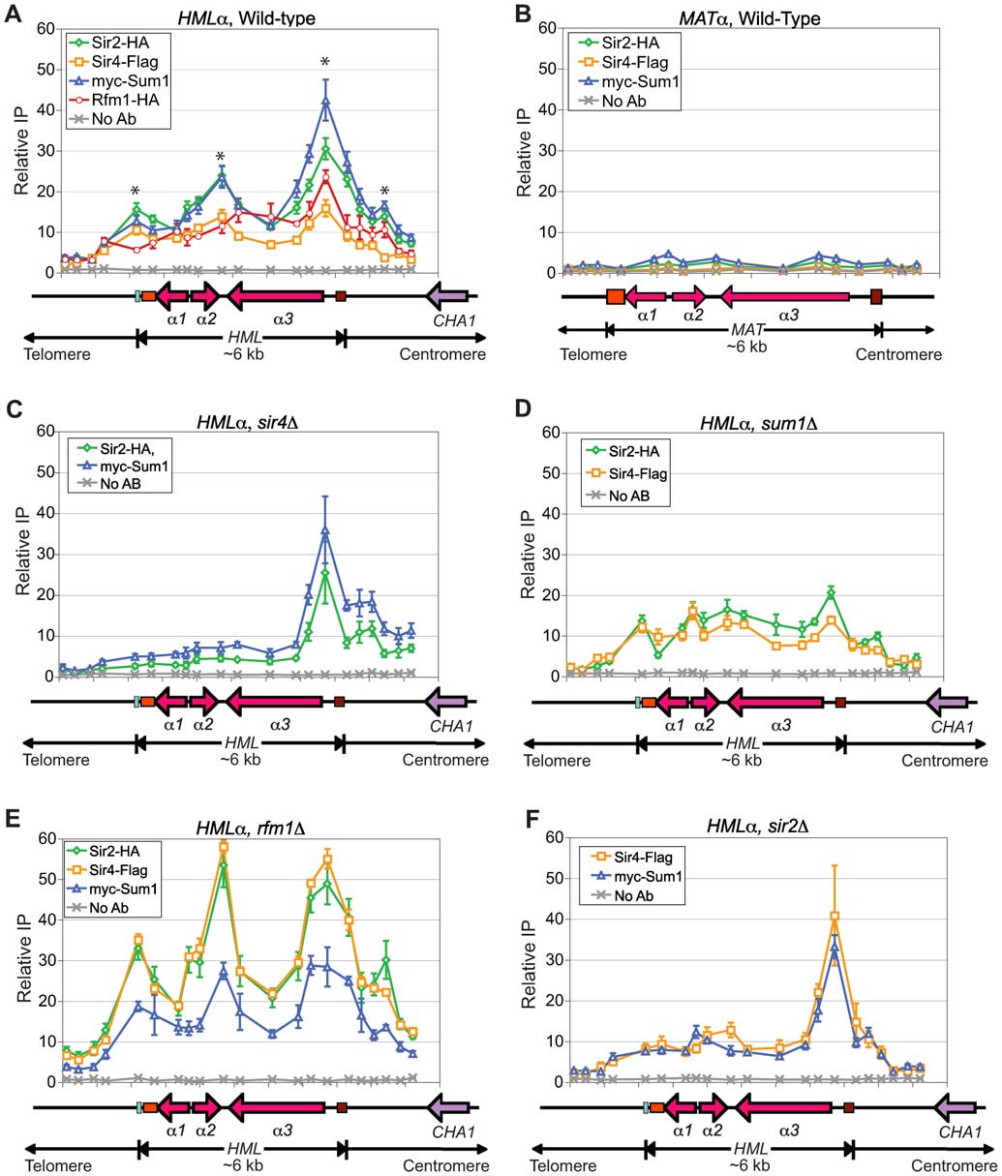
KlSir2, KlSir4, KlSum1, and KlRfma1 spread across *HMLα*. (A) The association of KlSir2-HA, KlSir4-Flag, myc-KlSum1 (LRY2239) and KlRfm1-HA (LRY2327) with *HMLα* as assessed by chromatin IP followed by quantitative PCR. The y-axis represents the relative enrichment normalized to a control locus, *RRP7*, which is not detectably associated with KlSir2, KlSir4 or KlSum1. A diagram of the *HMLα* locus is shown under the x-axis. The aqua bar represents the characterized silencer and the orange and brown boxes represent sequences found at *HMLα*, *MAT*, and *HMR* *a* loci. Asterisks indicate the peaks of enrichment. (B) The association of KlSir2-HA, KlSir4-Flag and myc-KlSum1 with the *MATα* locus in a strain in which the *α*-cassette is only found at *MAT* (LRY2398). (C) The association of KlSir2-HA and myc-KlSum1 with *HMLα* in a *sir4*Δ strain (LRY2281). (D) The association of KlSir2-HA and KlSir4-Flag with *HMLα* in a *sum1*Δ strain (LRY2158). (E) The association of KlSir2-HA, KlSir4-Flag and myc-KlSum1 with *HMLα* in a *rfm1*Δ strain (LRY2528). (F) The association of KlSir4-Flag and myc-KlSum1 with *HMLα* in a *sir2*Δ strain (LRY2388). All y-axes are set to the same scale to facilitate the comparison of protein associations in different experiments. Error bars represent the SEM.

Given the surprising result that KlSum1 affects the expression of *HMLα*, it was important to investigate whether KlSum1 acts directly at *HMLα* to silence transcription. We also examined the association of KlSir2 and KlSir4 with *HMLα*, as the association of these proteins with *HMLα* had not been assessed previously. We used chromatin immunoprecipitation to map the distributions of KlSir2, KlSir4, KlSum1 and KlRfm1 across *HMLα*. We observed a robust enrichment of all four proteins across the entire *HMLα* locus (Figure 3A), demonstrating that not only KlSir2 and KlSir4, but also the components of the SUM1 complex, KlSum1 and KlRfm1, spread across this locus. Therefore, KlSum1 contributes directly to transcriptional silencing at *HMLα*.

The enrichment of KlSir2, KlSir4 and KlSum1 peaked at a previously identified silencer ([32], represented as an aqua bar in Figure 3), suggesting that this sequence may stabilize the association of silencing proteins with chromatin. Three other peaks were also observed (indicated by asterisks in Figure 3A): one in the intergenic region in which the *α2* and *α3* genes converge, one in the *α3* promoter, and a smaller peak on the centromere proximal side of *HMLα*. These peaks could represent additional silencers or proto-silencers. Curiously, two of the peaks coincided with sequences that are conserved between the transcriptionally silent *HMLα* locus and the transcriptionally active *MATα* locus. If these peaks represent binding sites for silencing factors, then these factors might be recruited to *MATα*. To examine this possibility, we constructed a strain in which the *α*-cassette at *HML* was replaced with an **a**-cassette, so that the only *α*-cassette in the genome was at the *MAT* locus. Using this strain, we investigated whether KlSir2, KlSir4 or KlSum1 associated with the *MAT* locus. All three proteins associated with control loci (data not shown). However, we observed no significant enrichment of KlSum1, KlSir2 or KlSir4 anywhere along the *MATα* locus (Figure 3B). Therefore, the peaks of silencing proteins at the *α3* promoter and the *α2–α3* intergenic regions are specific to the *HMLα* locus, and these sequences cannot recruit silencing proteins independently.

### Both KlSir4 and KlSum1 recruit KlSir2 to *HMLα*

Sir2 deacetylases lack DNA-binding and histone-binding domains and consequently are recruited to chromatin through adaptor proteins such as Sum1, a DNA binding protein, or Sir4, a histone binding protein. To determine whether KlSir4 and/or KlSum1 recruit KlSir2 to *HMLα*, we examined the association of KlSir2 with *HMLα* in strains lacking these proteins. In a *sir4*Δ strain, the enrichments of KlSir2 and KlSum1 were significantly reduced over the silencer and across the open reading frames of *α1*, *α2*, and *α3* (Figure 3C). However, the associations of KlSir2 and KlSum1 with the promoter of *α3* and centromere-proximal side of *HMLα* were unchanged. Thus, there may be different requirements for the assembly of silenced chromatin on the two sides of the *HMLα* locus. On the telomere-proximal side, containing the known silencer, KlSir4 is important for the recruitment and spreading of silencing proteins. However, on the centromere-proximal side, the recruitment of KlSum1 and KlSir2 is independent of KlSir4.

The ability of KlSir2 to associate with the centromere-proximal side *HMLα* in the absence of KlSir4 suggests that another protein is recruiting KlSir2 to this region. To determine whether KlSum1 is required for the recruitment or spreading of KlSir2 and KlSir4, we examined the associations of these proteins with *HMLα* in a *sum1*Δ strain. The deletion of KlSum1 caused a reduction in the association of KlSir2 at the *α*2–*α*3 intergenic region, the *α*3 promoter and on the centromere-proximal side of the *HMLα* locus. There was no observable difference in the association of KlSir4 with *HMLα* (Figure 3D). These results suggest that KlSum1 is important for stabilizing the association of KlSir2 with the *HMLα* locus, particularly at the *α*3 promoter and centromere-proximal regions, but that it is not absolutely required for the recruitment or spreading of either KlSir2 or KlSir4. Together, these results indicate that neither KlSir4 nor KlSum1 is solely responsible for the recruitment of KlSir2 to *HMLα*. This finding is consistent with the independent interactions of KlSir2 with KlSir4 and KlSum1 (Figure 1).

The greater level of transcription of *HMLα* in a *sir2*Δ strain compared to an *rfm1*Δ strain (Figure 2) suggests that KlRfm1 is not critical for the recruitment of KlSir2 or other silencing proteins. In fact, in the absence of KlRfm1, all three silencing proteins, KlSir2, KlSir4 and KlSum1, still associated with the entire *HMLα* locus (Figure 3E). The enrichment of KlSum1 was indistinguishable between the wild-type and *rfm1*Δ strains, indicating that its association with *HMLα* does not require KlRfm1 and may be an inherent property of the Sum1 protein. Interestingly, the enrichments of both KlSir2 and KlSir4 were significantly enhanced in the *rfm1*Δ strain compared to the wild-type strain, although the overall pattern, with peaks of association at the silencer, *α2–α3* intergenic region, *α3* promoter and centromere-proximal side of *HMLα*, was maintained. Perhaps in the absence of KlRfm1, KlSir2 is better able to associate with KlSir4.

In *S. cerevisiae*, the deacetylase activity of Sir2 is required for the spreading of Sir3 and Sir4 [18],[19],[20]. To determine whether a similar requirement exists in *K. lactis*, we examined the associations of KlSir4 and KlSum1 with *HMLα* in a *sir2*Δ strain. KlSir4 and KlSum1 were reduced over the silencer and the three open reading frames (Figure 3F). However, both silencing proteins remained strongly associated with the *α3* promoter, and KlSir4 displayed a more robust enrichment with this region in the absence of KlSir2. This pattern of association is similar to the distribution of KlSum1 and KlSir2 in the *sir4Δ* strain (Figure 3C). Therefore, KlSir2 may contribute to the assembly of silenced chromatin on the telomere-proximal side of *HMLα*, but it is not required to assemble these factors at the *α3* promoter.

### KlSum1 associates with *HMLα* independently of KlSir2 and KlSir4

Given that KlSum1 is a DNA-binding protein, we were curious whether it binds directly to a sequence at *HMLα*. The mid-sporulation element (MSE) consensus sequence, to which Sum1 binds in *S. cerevisiae*, appears to be conserved in *K. lactis*, as it occurs at the promoters of a number of sporulation genes (data not shown). However, a match to the MSE consensus sequence was not found in the known telomere-proximal silencer (aqua box) or the rest of the *HMLα* locus. Moreover, the observation that the enrichment of KlSum1 was significantly reduced on the telomere-proximal side of *HMLα* in the absence of KlSir4 or KlSir2 (Figure 3C and 3F) makes it unlikely that KlSum1 binds directly to this side of the locus. Furthermore, KlSum1 did not associate with the *MATα* locus (Figure 3B), indicating that the sequences conserved between *MATα* and *HMLα* are unable to recruit KlSum1 directly. It remains possible that KlSum1 binds directly to a non-MSE sequence on the centromere-proximal side of the *HMLα*, and KlSum1 did associate with this region of *HMLα* in the absence of both KlSir2 and KlSir4 (Figure S3), indicating that the recruitment of KlSum1 to *HMLα* is independent of KlSir2 and KlSir4. However, it is also possible that another, unidentified protein recruits KlSum1 to this region.

### KlSir2 and KlSum1, but not KlSir4, repress *HMRa*

**Figure 4:**
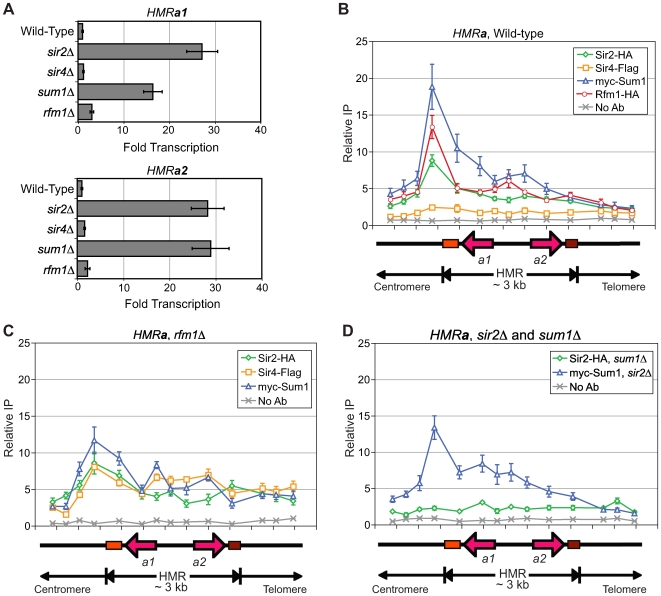
KlSir2 and KlSum1, but not KlSir4, silence and spread across *HMRa*. (A) Quantitative RT-PCR analysis of *HMR* *a* *1* and *HMR* *a* *2* in wild-type (SAY538), *sir2*Δ (SAY544), *sir4*Δ (LRY1946), *sum1*Δ (LRY1947), and *rfm1*Δ (LRY2529) strains. The fold induction was determined as for Figure 2. (B) The association of KlSir2-HA, KlSir4-Flag, myc-KlSum1 (LRY2285) and KlRfm1-HA (LRY2328) with *HMRa* as assessed by chromatin IP followed by quantitative PCR. (C) The association of KlSir2-HA (LRY2528), myc-KlSum1 and KlSir4-Flag (LRY2529) with *HMR* *a* in a *rfm1*Δ strain. (D) The association of KlSir2-HA with *HMR* *a* in a *sum1*Δ strain (LRY2126) and the association of myc-KlSum1 with *HMR* *a* in a *sir2*Δ strain (LRY2390). All y-axes are set to the same scale to facilitate the comparison of protein associations in different experiments. Error bars represent the SEM.

We next investigated the roles KlSir2, KlSir4, KlSum1 and KlRfm1 have in regulating the other cryptic mating-type locus, *HMR* ***a***. In *S. cerevisiae*, both *HM* loci are silenced by the same set of Sir proteins. However, in *K. lactis*, deletion of KlSir4 had little effect on the expression of the ***a1*** or ***a2*** genes found at *HMR* ***a*** (Figure 4A). Furthermore, deletion of KlAsf2, the paralog of KlSir4, either singly or in conjunction with KlSir4 did not result in derepression of *HMR* ***a*** (Figure S4). In contrast, deletion of KlSir2 or KlSum1 resulted in a substantial derepression of *HMR* ***a1*** and *HMR* ***a2***, whereas deletion of KlRfm1 resulted in very little change in *HMR* ***a1*** or *HMR* ***a2*** expression (Figure 4A). These results suggest that only a subset of the proteins that contribute to the silencing of *HMLα* also repress *HMR* ***a***.

To determine whether KlSir2 and KlSum1 act directly at *HMR* ***a***, we examined their association by chromatin immunoprecipitation. We observed an asymmetric distribution of KlSir2 and KlSum1, as well as KlRfm1, with the *HMR* ***a*** locus. A substantial peak of enrichment was observed on the centromere-proximal side of *HMR* ***a***, and a shoulder extended across the open reading frames (Figure 4B). The peak likely indicates the location of a silencer element. In contrast to KlSir2 and KlSum1, there was no significant association of KlSir4 with any part of *HMR* ***a***, consistent with the deletion of *SIR4* resulting in no change in the transcription of *HMR* ***a1*** and *HMR* ***a2***. These results indicate that KlSum1 and KlSir2, but not KlSir4, are responsible for repressing *HMR* ***a***. Thus, the mechanisms of silencing at *HMR* ***a*** and *HMLα* are distinct.

Curiously, KlRfm1 associated with *HMR* ***a*** (Figure 4B), yet was not required for repression of the *HMR* ***a1*** and *HMR* ***a2*** genes (Figure 4A). We examined the association of KlSum1 and KlSir2 with *HMR* ***a*** in a *rfm1*Δ strain and found that KlSum1 was only slightly reduced at the proposed silencer (Figure 4C). Intriguingly, KlSir2 was still able to associate with *HMR* ***a*** in the absence of KlRfm1, despite the fact that it no longer co-precipitated with KlSum1 (Figure 1A). We propose that the absence of KlRfm1 may enable KlSir4 to interact with KlSir2 and KlSum1, thereby stabilizing the association of KlSir2 with *HMR* ***a***. To test this hypothesis, we assessed whether KlSir4 associated with *HMR* ***a*** in an *rfm1*Δ strain, and indeed, KlSir4 associated with *HMR* ***a*** (Figure 4C). This result is reminiscent of the increase in KlSir4 at *HMLα* in the absence of KlRfm1 (Figure 3E).

To determine whether KlSum1 and KlSir2 depended on one another for association with *HMR* ***a***, we performed chromatin immunoprecipitation experiments in the absence of KlSum1 or KlSir2. In the absence of KlSum1, KlSir2 no longer associated with any region of the *HMR* ***a*** locus (Figure 4D), and therefore KlSum1 was required for recruitment of KlSir2 to *HMR* ***a***. This result contrasts with what was observed in the absence of KlRfm1 (Figure 4C). Deletion of KlSir2, like deletion of KlRfm1, resulted in a reduced association of KlSum1 with the proposed silencer at *HMR* ***a***. Despite this reduction, KlSum1 still spread across *HMR* ***a*** (Figure 4D). Thus, the association and spreading of KlSum1 does not require KlSir2 or KlRfm1.

### KlSir2 and KlSum1 repress mid-sporulation genes in a promoter-specific manner

**Figure 5:**
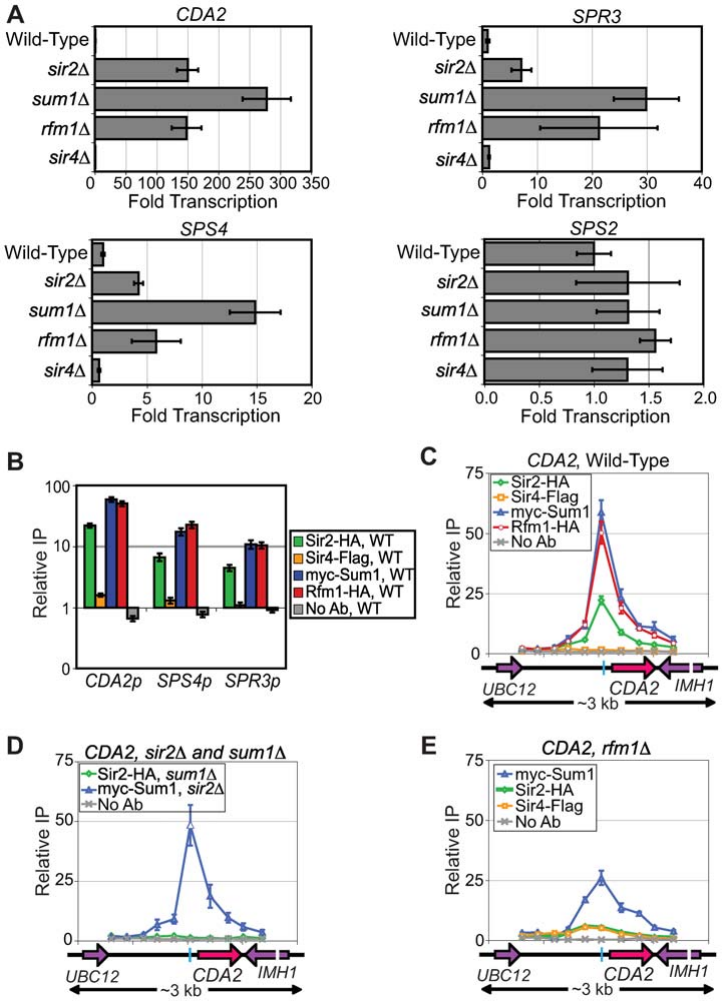
KlSir2, KlSum1, and KlRfm1 repress sporulation genes in a promoter-specific manner. (A) Quantitative RT-PCR analysis of *CDA2* (*KLLA0C17226g*), *SPS4* (*KLLA0F08679g*), *SPR3* (*KLLA0B08129g*) and *SPS2* (*KLLA0C01001g*) mRNA in wild-type (SAY538), *sir2*Δ (SAY544), *sum1*Δ (LRY1947), *rfm1*Δ (LRY2529) and *sir4*Δ (LRY1946) strains. The fold induction was determined as for Figure 2. (B) The association of KlSir2-HA, KlSir4-Flag, myc-KlSum1 (LRY2285) and KlRfm1-HA (LRY2328) with the promoters of *CDA2*, *SPS4* and *SPR3* was assessed by chromatin IP followed by quantitative PCR. The y-axis is a log-scale. (C) Distribution of KlSir2-HA, KlSir4-Flag, myc-KlSum1 (LRY2285) and KlRfm1-HA (LRY2328) across the *CDA2* locus. The blue bar in the schematic represents the conserved MSE sequence. (D) The association of KlSir2-HA with *CDA2* in a *sum1*Δ strain (LRY2126) and association of myc-KlSum1 with *CDA2* in a *sir2*Δ strain (LRY2390). (E) The association of KlSir2-HA and myc-KlSum1 with *CDA2* in a *rfm1*Δ strain (LRY2529). All y-axes are set to the same scale to compare changes in protein association across experiments. Error bars represent the SEM.

In *S. cerevisiae*, the Sum1-Hst1 complex represses mid-sporulation genes. To assess whether KlSir2 regulates mid-sporulation genes in a manner similar to ScHst1, we isolated RNA from wild-type, *sir2*Δ, *sum1*Δ and *rfm1*Δ strains and examined expression of the *K. lactis* orthologs of the mid-sporulation genes *CDA2*, *SPR3*, *SPS4*, and *SPS2* that are repressed by ScHst1 in *S. cerevisiae* [23]. Deletion of KlSir2, KlSum1 and KlRfm1 all resulted in derepression of *CDA2*, *SPS4*, and *SPR3*, but not *SPS2* (Figure 5A, note the different scales of the x-axes). We also examined whether KlSir4 has a role in regulating transcription of these genes, as KlSir2 and KlSum1 functioned with KlSir4 to regulate *HMLα*. However, the *sir4*Δ strain had no effect on the expression of *CDA2*, *SPS4*, *SPR3* or *SPS2* (Figure 5A). Therefore, KlSum1, KlSir2 and KlRfm1, repress sporulation genes independently of KlSir4. In addition, many (*CDA2*, *SPS4* and *SPR3*), but not all (*SPS2*) of the targets of the Sum1-Hst1 complex in *S. cerevisiae* are also targets in *K. lactis*.

To determine if KlSir2, KlSum1, and KlRfm1 repress mid-sporulation genes directly, we used chromatin immunoprecipitation to assess the association of KlSir2, KlSum1, KlRfm1 and KlSir4 with the promoters of these genes. KlSir2, KlSum1 and KlRfm1 were enriched at the promoters of *CDA2*, *SPS4* and *SPR3* (Figure 5B), suggesting that these proteins repress these genes directly, presumably as a complex. In contrast, KlSir4 did not associate with mid-sporulation genes, consistent with the *sir4*Δ strain having no effect on transcription. To address whether KlSir2, KlSum1 and KlRfm1 spread at sporulation genes, as they do at *HMLα* and *HMR* ***a***, we examined a 3-kb region around the *CDA2* promoter and open reading frame. A relatively narrow peak of KlSum1, KlRfm1 and KlSir2 coincided with an MSE consensus sequence at the promoter of *CDA2* (indicated by the blue bar in the schematic), and the association of these proteins diminished significantly in both directions (Figure 5C), suggesting that these proteins do not spread at the *CDA2* locus. Therefore, the ability of the SUM1 complex to spread differs between the *HM* loci and mid-sporulation genes.

We had observed at *HMLα* that KlAsf2 was antagonistic to silencing (Figure S2), and it was possible that KlAsf2 restricts the spreading of the Sum1-Sir2 complex at sporulation genes and therefore accounts for the difference in spreading at *HMR* ***a*** compared to sporulation genes. To test this hypothesis, we assessed the distribution of KlSum1 and KlSir2 at the sporulation gene *CDA2* in an *asf2*Δ strain. We observed no changes in the distribution of KlSir2 and KlSum1 across the *CDA2* locus (Figure S5A). Furthermore, the transcription of several mid-sporulation genes was not altered (Figure S5B). Therefore, KlAsf2 only antagonized silencing at *HMLα*.

We discovered that KlSir2 was more dependent on KlRfm1 for recruitment to *CDA2* as compared to *HMR* ***a***. At *HMR* ***a***, KlSir2 required KlSum1 but not KlRfm1 for recruitment (Figure 4C and 4D). In contrast, the association of KlSir2 with *CDA2* was greatly reduced in both *sum1*Δ and *rfm1*Δ strains (Figure 5D and 5E). This dependence was similar to what has been observed for the *S. cerevisiae* SUM1 complex at mid-sporulation genes. One potential explanation for the reduced role of KlRfm1 at the *HM* loci is the ability of KlSir4 to compensate for the loss of KlRfm1. For example, at both *HMLα* and *HMR* ***a***, the association of KlSir4 increased in the absence of KlRfm1 (Figure 3E and Figure 4C). In keeping with the greater role of KlRfm1 at *CDA2*, we observed only a modest increase in the association of KlSir4 (Figure 5D) in the absence of KlRfm1. We also found that the ability of KlSum1 to associate with the promoter of *CDA2* was unaltered in the absence of KlSir2 (Figure 5D), and was reduced, but not abolished, in the absence of KlRfm1 (Figure 5E). Thus, KlRfm1 contributes to the ability of the SUM1 complex to associate with DNA. We conclude that the promoter-specific mechanism by which the SUM1 complex represses mid-sporulation genes is conserved between *K. lactis* and *S. cerevisiae*.

### KlSum1 and KlSir2 also repress cell-type–specific genes

**Figure 6:**
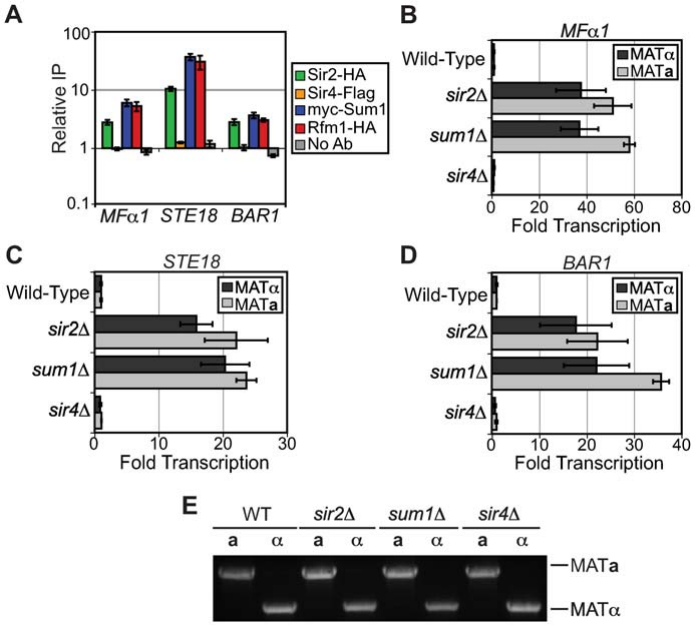
KlSir2 and KlSum1 repress cell-type–specific genes. (A) Association of KlSir2-HA, KlSir4-Flag, myc-KlSum1 (LRY2285) and KlRfm1-HA (LRY2328) at the *MFα1* (*KLLA0E19173g*), *STE18* (*KLLA0E06138g*) and *BAR1* (*KLLA0D15917g*) promoters in a *MATα* strain as assessed by chromatin IP followed by quantitative PCR. The y-axis is a log-scale. (B) Quantitative RT–PCR analysis of *MFα1* mRNA in *MATα* wild-type (SAY538), *sir2*Δ (SAY544), *sum1*Δ (LRY1947), and *sir4*Δ (LRY1946) strains and *MAT* *a* wild-type (CK213), *sir2*Δ (SAY569), *sum1*Δ (LRY2035) and *sir4*Δ (LRY2038) strains. (C) Quantitative RT-PCR analysis of *STE18* mRNA in the same strains analyzed in (B). (D) Quantitative RT-PCR analysis of *BAR1* mRNA in the same strains analyzed in panel B. Error bars represent the SEM. (E) PCR amplification of *MAT* loci in strains analyzed in (B–D) using mating-type specific primers.

The KlSum1-KlSir2 complex is clearly critical to the regulation of sexual identity and the sexual cycle as it represses both the *HM* loci and sporulation genes. However, the Sum1-Sir2 complex may have an even broader role in controlling sexual identity. It has recently been shown in both *Saccharomyces bayanus* and *S. cerevisiae* that Sum1 represses *α*-specific genes [24]. To investigate whether the Sum1-Sir2 complex in *K. lactis* also represses *α*-specific genes or other cell-type specific genes, we examined whether promoters of cell-type specific genes were associated with KlSir2. Remarkably some, but not all, *α*-specific, **a**-specific and haploid-specific genes were associated with KlSir2 (Figure 6A and data not shown). For example, the *α*-specific gene *MFα1*, the **a**-specific gene *BAR1*, and the haploid-specific gene *STE18* were associated with KlSir2, KlSum1, and KlRfm1, but not KlSir4 (Figure 6A).

To determine whether the Sum1-Sir2 complex represses these genes, RNA was isolated from both *MAT* ***a*** and *MATα* cells and expression of *MFα1*, *STE18*, and *BAR1* was examined by quantitative RT-PCR. *MFα1* encodes *α*-pheromone and in *S. cerevisiae* is expressed in *MATα* cells but not in *MAT* ***a*** cells. However in *K. lactis*, deletion of KlSum1 or KlSir2 resulted in the derepression of *MFα1* in both cell types to a comparable extent (Figure 6B). Quantification of cDNA from wild-type cells revealed that *MFα1* was repressed to a similar degree in both *MAT* ***a*** and *MATα* cells (Figure S6). These findings suggest that during vegetative growth, haploid *K. lactis* cells are not transcribing or producing *α*-pheromone, regardless of their mating-type identity, and that the Sum1-Sir2 complex contributes to the repression of this gene.

*STE18* encodes the G protein gamma subunit in the mating signaling pathway and in *S. cerevisiae* is expressed in both *MATα* and *MAT* ***a*** haploid cells. In *K. lactis*, *STE18*, like *MFα1*, was repressed in both *MATα* and *MAT* ***a*** cells (Figure S6), and deletion of either KlSir2 or KlSum1 resulted in derepression of *STE18* in both cell types (Figure 6C). *BAR1* encodes an *α*-pheromone protease that in *S. cerevisiae* is expressed to a greater extent in *MAT* ***a*** than *MATα* cells. This pattern of gene expression was also found in *K. lactis* (Figure S6). However, as for *MFα1* and *STE18*, deletion of KlSum1 or KlSir2 resulted in the derepression of *BAR1* in both *MAT* ***a*** and *MATα* cells (Figure 6D). To verify that we had correctly identified the mating-type of the strains used for these experiments, we analyzed a segment of the *MAT* locus using mating-type specific PCR primers that yield different sized products in *MAT* ***a*** and *MATα* strains. All strains had the expected genotypes (Figure 6E). Together, these results suggest that the KlSum1-KlSir2 complex represses a variety of cell-type specific genes as well as mid-sporulation genes and the *HM* loci. Therefore, this complex represents an important regulator of yeast sexual identity and activity.

## Discussion

### The Sum1-Sir2 complex employs multiple mechanisms to repress transcription

**Figure 7:**
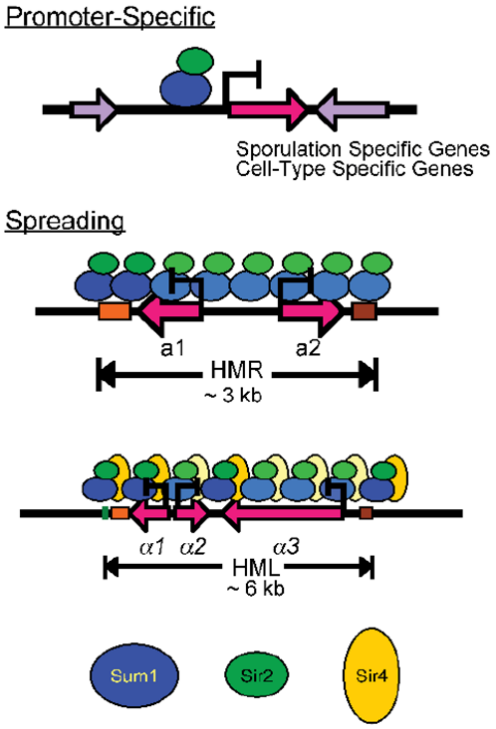
Mechanisms of repression mediated by KlSum1-KlSir2. The KlSum1-KlSir2 complex participates in multiple mechanisms of repression: a promoter-specific mechanism that represses mid-sporulation and cell-type specific genes (top) as well as a long-range spreading mechanism that silences the cryptic mating-type loci either with Sir4 (*HMLα*; bottom) or without Sir4 (*HMR* *a*; middle). The darker shaded proteins represent stronger association than the lighter colored proteins.

This study has made the striking discovery that the Sum1-Sir2 complex in *K. lactis* achieves repression through several distinct mechanisms (Figure 7). In *S. cerevisiae*, the Sum1-Hst1 complex functions primarily as a promoter-specific repressor of mid-sporulation, *α*-specific, and NAD^+^-biosynthetic genes, and loss of ScSum1 or ScHst1 do not alter the expression of the *HM* loci [6],[33]. In contrast, in *K. lactis*, the Sum1-Sir2 complex not only uses a promoter-specific mechanism to repress the same sets of genes as in *S. cerevisiae* (Figure 7, top panel), it also has a major role in silencing the *HM* loci by forming extended chromatin structures (Figure 7, middle and lower panels).

Interestingly, the KlSum1-KlSir2 complex acts differently at *HMLα* (lower panel), where it works in conjunction with KlSir4, compared to *HMR* ***a*** (middle panel), where KlSir4 is not normally present. Thus, the mechanism by which *HMR* ***a*** is silenced is unlike the mechanism employed at *HMLα*. The absence of KlSir4 at *HMR* ***a*** is surprising, as the spreading of silencing proteins is thought to require a histone-binding protein, such as KlSir4, and neither KlSum1 nor KlSir2 is known to have this capacity. An important subject for future studies will be to determine how the spreading capacity of the KlSum1-KlSir2 complex is modulated at different genomic locations. It is possible that factors associated with the *HM* loci promote the spreading of KlSum1-KlSir2. For example, silencers may recruit additional proteins that facilitate the spreading process. We have recently found that the *HMR-E* silencer in *S. cerevisiae* can promote the assembly of silenced chromatin through a mechanism that is independent of recruitment [36], and it is possible that silencers in *K. lactis* have similar properties. Alternatively, factors associated with the promoters of mid-sporulation genes may limit or disable the spreading of KlSum1-KlSir2.

This study also revealed that, although the KlSum1-KlSir2 and KlSir4-KlSir2 complexes cooperate at *HMLα*, they have distinct contributions to chromatin assembly and transcriptional repression. For example, the KlSir4-KlSir2 complex was critical for assembly of silencing proteins on the telomere proximal side of *HMLα*. However, silenced chromatin on the centromere-proximal side did not depend on KlSir2 or KlSir4, but was affected by the loss of KlSum1. These results suggest that the chromatin structure differs on the two sides of *HMLα*, perhaps due to different types of silencer elements. Another indication that the KlSum1-KlSir2 and KlSir4-KlSir2 complexes have independent properties is the observation that the associations of KlSir4 and KlSir2 increased at *HMLα* and *HMR* ***a*** in the absence of KlRfm1. This result suggests that KlSir4 and KlRfm1 may compete for association with KlSir2.

### Association of silencing factors does not correlate with transcriptional activity at *HMLα*

One puzzling observation was that the absence of KlSir4 resulted in a relatively modest induction of the *HMLα1* and *HMLα2* genes despite a significant decrease in the associations of both KlSir2 and KlSum1 with the α1–α2 promoter. Conversely, the absence of KlSum1 resulted in a large increase of transcriptional activity yet had seemingly little effect on the associations of KlSir2 and KlSir4 with *HMLα*. These results are reminiscent of observations that, in some situations, Sir proteins in *S. cerevisiae* associate with *HM* loci but do not achieve repression [37],[38]. We speculate that the presence of the KlSum1-KlSir2 complex at *HMLα* is more critical for repression than is the presence of KlSir4. Moreover, KlSum1 and KlSir2 must be able to achieve repression over a distance, because their presence at the *HMLα3* promoter is sufficient to repress the *HMLα1* and *HMLα2* genes. Similarly, KlSum1 and KlSir2 may act at distance at *HMR* ***a***, as their greatest enrichment is some distance from the promoter. In contrast, the KlSir4-KlSir2 complex appears to be somewhat permissive to transcription in the absence of KlSum1. Perhaps this chromatin structure serves another biological function, such as preventing illegitimate mating-type switching. While *K. lactis* is considered to be a homothallic yeast species [39], an ortholog of the HO endonuclease, which initiates switching in *S. cerevisiae*, has not been identified [40], and mating-type switching presumably occurs through spontaneous homologous recombination. These switching events are relatively rare had have not been studied recently [39].

### *SIR2* and *HST1* subfunctionalized after duplication

This study was initiated to investigate how the deacetylases *SIR2* and *HST1* diverged after duplication. Two models, subfunctionalization and neofunctionalization, have been proposed to explain how duplicated genes diverge. We used the non-duplicated KlSir2 as a proxy for the ancestral protein and found that it interacted with both KlSir4 and KlSum1 (Figure 1), the partners of ScSir2 and ScHst1, respectively. Furthermore, KlSir2 functioned as a promoter-specific repressor of sporulation genes (similar to ScHst1; Figure 5) and also as a silencing factor that spreads across the *HM* loci (similar to ScSir2; Figure 2, Figure 3, Figure 4). Therefore, KlSir2 has both Hst1- and Sir2-like functions. The most parsimonious interpretation of these results is that the ancestral deacetylase also had both functions and that subfunctionalization occurred after duplication. This conclusion is supported by the observation that ScSir2 has retained the ability to substitute for ScHst1 in its absence [26]. This is an important contribution to the understanding of the evolution of duplicated genes, as it provides an example of subfunctionalization of protein-protein interactions as opposed to partitioning of expression patterns, which have previously been documented [41].

Previous work provides insight into how the subfunctionalization of *SIR2* and *HST1* occurred. A chimeric protein consisting of the N-terminus of ScSir2 and the C-terminus of ScHst1 has both Sir2- and Hst1-like functions in *S. cerevisiae* [26],[42]. This observation suggests that different regions of the deacetylases are important for specifying interactions with the SIR and SUM1 complexes. It is likely that the ancestral deacetylase used these same domains to interact with the SIR and SUM1 complexes. After *SIR2* was duplicated, the two copies likely acquired mutations that reduced their affinities for either the SIR or SUM1 complexes, leading to subfunctionalization.

Over the course of evolution it was not simply the deacetylase that subfunctionalized. The proteins associated with Sir2 and Hst1 are used in different ways to achieve repression of essentially the same sets of genes in *S. cerevisiae* and *K. lactis*. Other studies have revealed changes in the transcriptional regulatory circuits of yeasts [13],[43],[44]. However in previous examples, evidence suggested that promoter elements have changed to bring genes under the control of different regulators or alter their expression patterns. This study expands the scope of adaptations that can lead to modifications in transcriptional networks, as it reveals that the molecular mechanisms by which regulatory proteins act can also change over evolutionary time.

### Evolution of *SIR4* and *ASF2* genes

In addition to the paralogs *SIR2* and *HST1*, we investigated a second duplicated gene pair, *SIR4* and *ASF2*. *SIR4* and *ASF2* were tandemly duplicated prior to the whole genome duplication and to the divergence of *Kluyveromyces* and *Saccharomyces* species. Due to their tandem arrangement and rapid rate of sequence change, it has been difficult to determine which gene is the ortholog of *ScSIR4* or *ScASF2*. Functional analysis shows that *KLLA0F14320g* silences *HMLα* (Figure 2, Figure 3, and [31]) as thus has a Sir4-like function , whereas *KLLA0F13998g* antagonizes silencing at *HMLα* (Figure S2) and thus has Asf2-like function. This experimental evidence seems to contradict phylogenetic analyses implying that *KLLA0F13998g* is the ortholog of *ScSIR4*, as it clusters with *SIR4* genes from other yeast species, and that *KLLA0F13420g* is an ortholog of *ScASF2*, as it clusters with *ASF2* genes as well as *SIR4* genes from *Candida glabrata*, *S. castellii*, *S. kluyveri* and *Ashbya gossypii* (Figure S1 and [7]). However, this gene tree does not match the species phylogeny, perhaps due to the rapid rate of sequence change and consequently may not accurately reflect the evolutionary relationships among these genes.

### The *SUM1-1* mutation in *S. cerevisiae*

The observation that KlSum1 spreads at the *HM* loci provides a new perspective on the perplexing *SUM1-1* mutation identified in *S. cerevisiae*. This mutation was originally isolated as a suppressor of a *sir2*Δ mutation [45] and results from a single point mutation, T988I. It causes Sum1 to re-localize from mid-sporulation promoters to the *HM* loci and form an extended chromatin structure [46],[47]. It had been thought that the *SUM1-1* mutation is a gain-of-function mutation that creates the ability to spread *de novo*, and it was surprising that a single amino acid change could have such a profound effect. However, this study suggests a new interpretation. The ability of both KlSum1 and ScSum1-1 to spread at *HM* loci suggests that the ancestral Sum1 also had this ability, which was subsequently lost in the *Saccharomyces* lineage. Consequently, wild-type ScSum1 probably retains most of the properties necessary to spread, and the T988I mutation unmasks this hidden potential.

Our knowledge of the mechanism of the *SUM1-1* mutation may provide insights into how the spreading of KlSum1 is controlled. Residue T988 of ScSum1 is conserved in KlSum1, as well as in many other budding yeasts, and is located in the DNA-binding domain. Mutating this residue reduces the affinity of Sum1 for DNA [48] and replacing threonine 988 with isoleucine enables the protein to associate with new partners - ORC (the Origin Recognition Complex) and itself [47],[48],[49]. These observations led to the hypothesis that the *SUM1-1* mutation occurs in an interaction domain, and the switch between threonine and isoleucine causes the protein to interact with different partners [48]. Perhaps this domain of KlSum1 also has the capacity to interact with multiple partners, and the genomic context dictates whether this surface functions as a DNA-binding domain to recruit the Sum1-Sir2 complex to mid-sporulation genes or as a self-associating surface to enable KlSum1 to propagate along the chromatin at the *HM* loci.

### The Sum1-Sir complex as a master regulator of the yeast sexual cycle

The *K. lactis* Sum1-Sir2 complex plays a critical role as a regulator of sexual identity because it regulates some cell-type specific genes (Figure 6). Within budding yeasts there has been a transition from positive to negative regulation of **a**-specific genes. *Candida albicans* requires an activator to turn on **a**-specific genes in *MAT* ***a*** cells, whereas in *S. cerevisiae*, **a**-specific genes are on by default and must be turned off in *MATα* cells [50]. *K. lactis* has been proposed to have an intermediate circuitry in regulating cell-type identity [43], as **a**-specific gene promoters share features of both *C. albicans* and *S. cerevisiae* promoters. In this study we have demonstrated that many cell-type specific genes, including **a**- and *α*-specific genes are repressed by the KlSum1-KlSir2 complex in both haploid cell types providing an additional level of regulation to sexual identity.

Differences between the life cycles of *K. lactis* and *S. cerevisiae* may heighten the importance of the Sum1-Sir2 complex in *K. lactis*. Vegetative growth of *K. lactis* occurs predominantly in the haploid phase, and mating occurs in response to nutrient deprivation, leading almost immediately to sporulation [39],[51],[52]. In contrast, *S. cerevisiae* propagates primarily in the diploid phase. Mating occurs shortly after germination in rich nutrient conditions, but sporulation of the resulting diploid cells is delayed until nutrients become scarce. Thus, unlike *S. cerevisiae*, *K. lactis* requires a mechanism to suppress mating of haploid cells under nutrient-rich conditions, and perhaps the Sum1-Sir2 complex contributes to this regulation by repressing some of the *α*-specific, **a**-specific, and haploid-specific genes required for mating. The use of a repressive complex containing an NAD^+^-dependent deacetylase may help connect the sexual cycle of *K. lactis* with nutrient availability.

## Materials and Methods

### Yeast media and methods

All *K. lactis* strains used in this study were grown at 30° in YPD medium containing 1% yeast extract, 2% peptone and 2% glucose. Antibiotic supplements were added to YPD medium at 50 µg/ml of clonNAT and 200 µg/ml of geneticin. Electroporation conditions were as described [53] with the following changes. Cells were washed with LiAc buffer (10 mM Tris pH 7.5, 270 mM sucrose, 1 mM lithium acetate) after initial centrifugation. After treatment with the pre-treating buffer (YPD, 20 mM HEPES pH 8.0, 25 mM DTT), cells were resuspended in LiAc buffer to a final concentration of 2×10^9^ cells/ml and electroporation was performed in a 0.2 cm cuvette, with a final at volume between 50 and 55 µl. The settings for electroporation were 1,000 V, 25 µF and 300 Ω. Cells transformed with antibiotic resistance markers were grown at 30° in YPD for 3–5 hours before being plated on selective medium.

Mating was carried out by mixing equal volumes of overnight cultures of the two parental strains, plating 4–10 µl on malt extract (ME) medium (2% malt extract, 2% agar) and incubating at 30° for 2–3 days. Cells were then streaked on media to select for diploids and subsequently transferred to ME plates for sporulation. After 3–4 days, the sporulated culture was suspended in 500 µl water, incubated at 56° for 15 minutes, and plated on media to select for alleles of interest. Genotypes were confirmed by PCR.

### Yeast strains

Strains used in this study were derived from SAY538 (Table S1). The *sir2*Δ::*KanMX* allele was obtained from S. Astrom. The *sir2*Δ*::NatMX*, *sir4*Δ*::URA3*, *asf2*Δ*::NatMX*, *sir4*Δ *asf2*Δ*::URA3*, *sum1*Δ*::NatMX* and *rfm1*Δ::*URA3* alleles were complete deletions of the open reading frames generated by one-step gene replacement. The replacement markers *NatMX* and *URA3* were derived from pAGT100 [54] and pRS316 [55], respectively. The *HMLa* allele was a fortuitous gene conversion event that occurred during the course of crossing a *sir2*Δ strain. The *SIR2*-HA, *RFM1*-HA and *SIR4*-Flag alleles were constructed by integrating the tag plus a selectable marker at the end of the open reading frame. Tagging cassettes were generated from pAGT105 [54] containing the HA-epitope tag along with the entire open reading frame of *NatMX* or p3FLAG-KanMX, [56] containing the Flag tag plus *KanMX*. The myc-*SUM1* allele was generated in two steps. First, a myc-*URA3*-myc-*SUM1* construct, derived from p3MPY-3xMyc, [57] was integrated into the *K. lactis* genome. After correct integration was confirmed by PCR, cells were grown in non-selective media to allow for recombination between the identical myc-tags and cells were plated on 5-FOA to select for the loss of the *URA3* marker. In all cases, the correct integration was confirmed by PCR using primers flanking the sites of recombination. To confirm that the tagged proteins were functional, expression of genes regulated by these factors was examined by quantitative RT-PCR. Alleles were moved into various genetic backgrounds (as described in Table S1) through genetic crosses.

### Gene expression analysis

RNA was isolated from logarithmically growing cultures of each strain using a hot phenol method [58]. Removal of DNA was as previously described [26]. To verify that there was no contaminating DNA, 1 µl of DNAse-treated material was used in a PCR reaction containing primers to amplify the *KlACT1* transcript. 1 µg of DNA-free RNA was used for cDNA synthesis as previously described [26]. To quantify the relative amounts of mRNA transcripts, approximately 0.025 µg of cDNA was analyzed by real-time PCR in the presence of SYBR Green using a Bio-Rad iCycler. The standard curve was generated with genomic DNA isolated from the wild-type strain (SAY538). Oligonucleotide sequences are provided in Table S2. Data were analyzed with iCycler iQ Optical System Software. Transcript levels of queried genes were first normalized to the *KlACT1* mRNA for each genetic background. The fold-induction was calculated by normalizing to the wild-type strain. Results represent the average fold induction (relative to wild-type) of at least two independent cultures of each strain background. The standard error measurement (SEM) was calculated from the differences in fold induction of two or more independent cultures from the mean.

### Chromatin immunoprecipitation

Chromatin immunoprecipitation was performed by harvesting approximately 50 OD (7×10^8^) of logarithmically growing cells, collected at an OD_600_ = 1.4. Cells were collected, washed twice in PBS, re-suspended in DMA (10 mM dimethyl adipimidate, 0.1% DMSO, 1× PBS) and rocked at room temperature for 60 minutes to crosslink. Subsequent to crosslinking, cells were washed twice with PBS, re-suspended in 36 ml PBS and rocked with 1% formaldehyde at room temperature for 60 minutes. The preparation of soluble chromatin and immunoprecipitation was performed as previously described [26]. Chromatin IP samples were analyzed by qPCR using a standard curve prepared from input DNA. The amounts of the immunoprecipitated DNA at experimental loci and a control locus, *KlRRP7*, were determined relative to the input DNA, and the relative enrichment of the experimental loci compared to the control locus was calculated. Oligonucleotide sequences are provided in Figure S7 and Table S3. Results represent the relative immunoprecipitation of two or more independent cultures of each strain background, and the SEM was calculated from differences in the relative enrichment from the mean. No antibody control data represent the average values from multiple chromatin IP experiments using different strains.

### Co-immunoprecipitations

Co-immunoprecipitations were performed by harvesting approximately 30 OD (4.2×10^8^) of logarithmically growing cells. The preparation of whole-cell lysates was performed as previously described [26]. Whole-cell lysates were incubated overnight at 4°C with 5 µl of α-HA (Sigma H-6908), α-Flag (Sigma F-7425) or α-myc (Millipore 06-549) antibody. Subsequently, 60 µl of Protein A agarose beads were added and samples were rotated at 4° overnight and protein was eluted in 75 µl 3× protein sample buffer (30% glycerol, 15% β-mercaptoethanol, 0.006% bromophenol blue, 0.1875 M Tris pH 6.8) for 3 minutes at 95°. 20 µl of IP samples and 7.5 µl of whole-cell extracts were electrophoretically fractionated on 7.5% polyacrylamide-SDS gels, transferred to nitro-cellulose membranes, and probed with either mouse polyclonal α-HA antibody (Sigma H-3663), mouse polyclonal α-myc antibody (Calbiochem OP10), rabbit (Sigma F-7425) or mouse (Sigma F-3165) α-Flag antibodies and detected by chemiluminescence (GE RPN2135).

## Supporting Information

Figure S1 Phylogenetic gene tree of *SIR4* and *ASF2* orthologs from several hemiascomycete species. Sequences and nomenclature were obtained from the Yeast Gene Order Browser (YGOB) (Byrne and Wolfe 2005) and analyzed using MEGA (Tamura et al 2007) to construct the neighbor joining gene tree. Bold, red font indicate species that underwent the whole genome duplication. *Sc = S. cerevisiae*, *Sb = S. bayanus*, *Cg = C. glabrata*, *Scas = S. castelli*, *Kp = K. polysporus*, *Zr = Z. rouxii*, *Ag = A. gossypii*, *Sk = S. kluyveri*, *Kt = K. thermotolerans*, *Kw = Kwaltii*. Common names, as notated in the YGOB, are given along with the systematic names in parantheses. *K.lactis* common gene names are not given to illustrate how *KLLA0F1430g* and *KLLA0F13998g* cluster.
(0.89 MB EPS)

Figure S2 Quantitative RT–PCR analysis of *HMLα1*, *HMLα2*, and *HMLα3* mRNA in wild-type (CK213), *sir2Δ* (SAY569), *sir4Δ* (LRY2038), *asf2Δ* (LRY2377), *asf2Δ sir4Δ* (LRY2374), and *sir2Δ asf2Δ* (LRY2523) strains. The amount of cDNA was first normalized to the control locus *ACT1*. The values shown here represent the relative amount of cDNA for each deletion strain compared to the wild-type strain. The data for wild-type *sir2Δ* and *sir4Δ* strains is the same as Figure 2. Error bars represent the SEM.
(0.65 MB EPS)

Figure S3 The association of myc-KlSum1 with *HMLα* as assessed by chromatin IP followed by quantitative PCR in a *sir2Δ sir4Δ* strain (LRY2530). The y-axis represents the relative enrichment normalized to a control locus, *RRP7*, which is not detectably associated with KlSir2, KlSir4 or KlSum1. Error bars represent the SEM. A schematic of the *HMLα* locus is shown under the x-axis.
(0.97 MB EPS)

Figure S4 Quantitative RT–PCR analysis of *HMRa1* and *HMRa2* in wild-type (SAY538), *sir4Δ* (LRY1946), *asf2Δ* (LRY1856), and *asf2Δ sir4Δ* (LRY1948) strains. The amount of cDNA was first normalized to the control locus *ACT1*. The values shown here represent the relative amount of cDNA for each deletion strain compared to the wild-type strain. The data for wild-type and *sir4Δ* strains is the same as Figure 4. Error bars represent the SEM.
(0.61 MB EPS)

Figure S5 (A) Quantitative RT–PCR analysis of *CDA2*, *SPS4*, *SPR3*, and *SPS2* mRNA in wild-type (SAY538) and *asf2Δ* (LRY1856) strains. The amount of cDNA was first normalized to the control locus *ACT1*. The values shown here represent the relative amount of cDNA for each deletion strain compared to the wild-type strain. The data for the wild-type strain is the same as Figure 5. (B) Association of KlSir2-HA and myc-KlSum1 with CDA2 as assess by chromatin IP followed by quantitative PCR in an *asf2Δ* strain (LRY2525). Error bars represent the SEM.
(1.00 MB EPS)

Figure S6 Quantitative RT–PCR analysis of the cryptic mating-type loci, mid-sporulation genes and cell-type–specific genes in wild-type strains of *MATa* and *MATα* cells. The amount of cDNA was normalized to *ACT1*.
(0.61 MB EPS)

Figure S7 Schematics of *HMLα*, *MATα*, *HMRa*, and *CDA2* with primer sets shown for chromatin IP quantitative PCR.
(2.82 MB EPS)

Table S1 *K. lactis* strains used in this study.
(0.03 MB DOC)

Table S2 Oligonucleotides used for quantitative RT–PCR.
(0.02 MB XLS)

Table S3 Oligonucleotides used for quantitative chromatin IP.
(0.05 MB XLS)
